# Torsion of a Luteal Cyst at 36 Weeks of Gestation: A Case Report

**DOI:** 10.7759/cureus.104960

**Published:** 2026-03-09

**Authors:** Platon Machavariani, Nickolas Kintraia, Natia Pkhaladze, George Burkadze, Maia Rizvadze, Nato Metskhvarishvili, Nino Khotivari, Besik Japaridze, Patman Tsaava, Eka Shvelashvili, Lali Barbakadze, Ketevan Chichua

**Affiliations:** 1 Department of Perinatology, The First University Clinic of Tbilisi State Medical University, Tbilisi, GEO; 2 Department of Obstetrics and Gynecology, Tbilisi State Medical University, Tbilisi, GEO; 3 Department of Obstetrics and Gynecology, The First University Clinic of Tbilisi State Medical University, Tbilisi, GEO; 4 Department of Pathology, Tbilisi State Medical University, Tbilisi, GEO; 5 Department of Obstetrics and Gynecology, Chachava Clinic, Tbilisi, GEO

**Keywords:** enlarged ovaries, lutein cysts, lutein cysts in pregnancy, ovarian torsion, third trimester of pregnancy

## Abstract

Ovarian torsion in the third trimester of pregnancy is rare and may contribute to maternal and fetal morbidity. The pathogenesis of this condition is not fully understood. Physiological changes in the ovaries during pregnancy usually suppress ovarian function. However, factors such as increased ovarian size and an elongated anatomical pedicle, which are not typical physiological changes of pregnancy, may predispose to torsion. Therefore, ovarian torsion can be more difficult to diagnose during pregnancy. It may be misdiagnosed as other conditions, and the differential diagnosis includes acute appendicitis, ruptured corpus luteum cyst, and urinary obstruction.

Here, we report the case of a G2P0 woman at 36 weeks of gestation who presented with lumbar and lower abdominal pain, difficulty urinating, urinary retention, and general weakness. Physical examination, laboratory tests, ultrasonography, and MRI were performed. A diagnosis of acute abdomen was made, and surgical intervention was successfully performed.

## Introduction

Torsion of the ovary on its pedicle results in infarction and hemorrhagic necrosis of the ovarian parenchyma due to obstruction of arterial, venous, and lymphatic flow [1]. Although the exact pathophysiology remains unclear, predisposing factors include increased ovarian size and an elongated pedicle [2]. Ovarian torsion is less common and more difficult to diagnose during pregnancy because of the anatomical and physiological changes associated with gestation. It may be misdiagnosed as other conditions, such as acute appendicitis, a ruptured corpus luteum cyst, or urinary obstruction [3-5].

## Case presentation

A 31-year-old pregnant woman at 36 weeks of gestation, G2P0, presented to the emergency department with complaints of pain in the lumbar area and the lower abdomen, difficulty urinating, urinary retention, and general weakness. According to the patient, the symptoms started 2 days ago with periodic dull pain in the lumbar region radiating to the abdominal wall. On the day of admission to our hospital, the patient developed difficulty urinating, and the pain in the lumbar and abdominal area became severe and constant; therefore, she was brought to our emergency department. The patient’s history revealed chickenpox, tonsillectomy, and pyelonephritis during childhood. This was her second pregnancy, and she had one miscarriage in the past. Her BMI was 23.1 kg/m².

At admission, clinical, laboratory, and ultrasonographic examinations were performed. General appearance: the patient is alert and oriented, appears mildly uncomfortable due to pain, but is not in acute distress. Skin: no pallor, jaundice, or cyanosis. A rash was observed on the face, abdomen, right shoulder, and arm. Hypertrichosis was observed on the abdominal wall and face. Vital signs at admission were blood pressure (BP) 125/75 mmHg, pulse (P) 74 bpm, respiratory rate (RR) 20/min, oxygen saturation (SpO₂) 97% on room air, and temperature (T) 36.6°C.

Abdominal examination revealed an enlarged abdomen consistent with 36 weeks of gestation, with no scars; hypertrichosis was observed on the abdominal wall. The abdomen was soft and non-tender, with pain in the hypogastric region mainly on the right side, without guarding or rigidity. The uterus was enlarged, corresponding to approximately 36 weeks of gestation; fundal height was 36 cm, fetal lie was longitudinal, and presentation was cephalic, with no uterine tenderness or contractions during examination. The fetal heart rate by cardiotocography (CTG) was 150-155 bpm. No palpable masses were detected apart from the gravid uterus. Pasternatsky’s sign was negative bilaterally.

Pelvic examination revealed normal external genitalia, with no vaginal bleeding or abnormal discharge. The cervix was closed and posterior, with no evidence of labor or membrane rupture. The right adnexa was painful during examination. There were no significant findings in the laboratory results (Table 1).

**Table 1:** Laboratory findings WBC: white blood cells; LYMPH: lymphocytes; NEU: neutrophils; RBC: red blood cells; HGB: hemoglobin; HCT: hematocrit; PLT: platelets; CRP: C-reactive protein

Laboratory findings			
Test	Result	Unit	Normal range
WBC	9.11	10⁹/L	4.00-11.00
LYMPH	19.4	%	20.0-45.0
NEU	70.60	%	50.00-70.00
RBC	3.32	10¹²/L	3.80-5.40
HGB	9.1	g/dL	12.5-15.5
HCT	27.0	L/L	36.0-45.0
PLT	234	10³ µL	150-380
CRP	16	mg/L	<5

**Figure 1:**
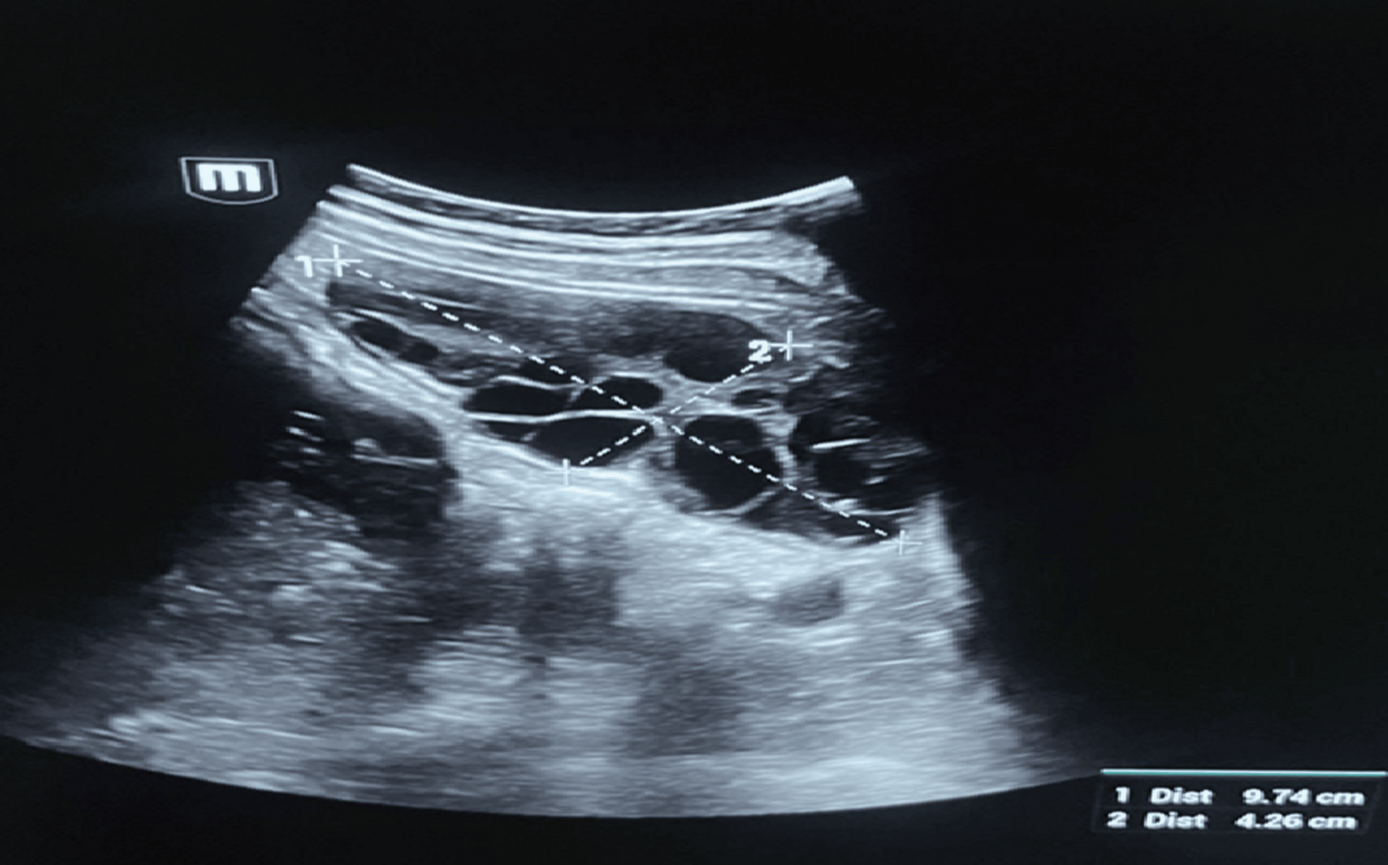
Ultrasound image of the enlarged ovary

The ultrasound examination was performed in the presence of pronounced meteorism (intestinal gas). However, near the lower margin of the right liver lobe, an oval-shaped, slightly nonhomogeneous fluid-tissue-like structure measuring 97 mm × 58 mm was identified, most likely corresponding to the right ovary. Ovarian blood flow was not clearly demonstrated on color Doppler. In the left upper quadrant of the abdominal cavity, a well-defined oval-shaped structure with multiple cystic inclusions measuring approximately 97 mm × 42 mm was identified, most likely corresponding to the left ovary. Ovarian blood flow was present on color Doppler. A small amount of adjacent fluid was also noted. Ultrasound of the urinary system revealed a dilated renal pelvis measuring 17 mm on the right and 15 mm on the left. No other significant findings were noted on ultrasound (Figure 1).

**Figure 2:**
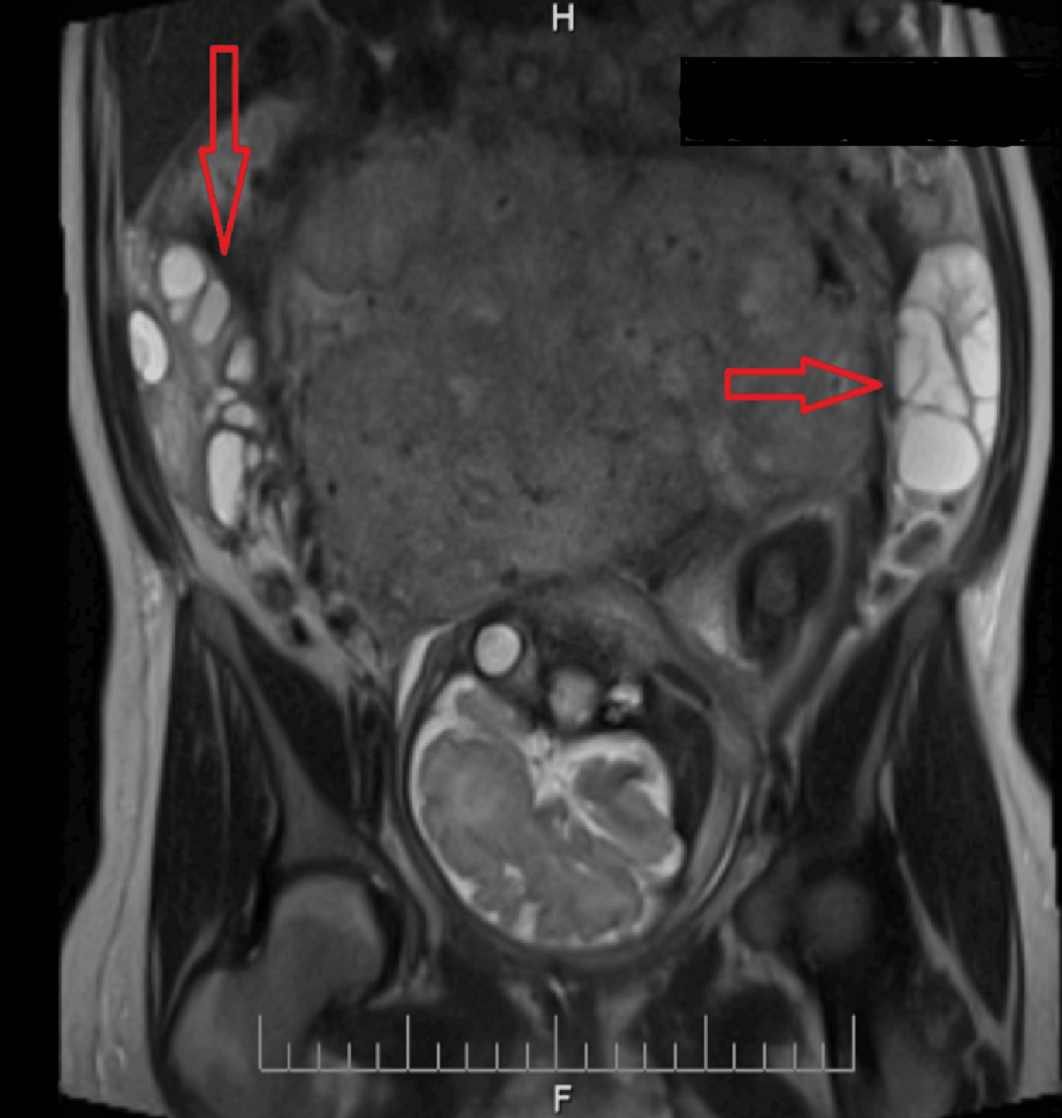
MRI image of enlarged ovaries H: head; F: foot

**Figure 3:**
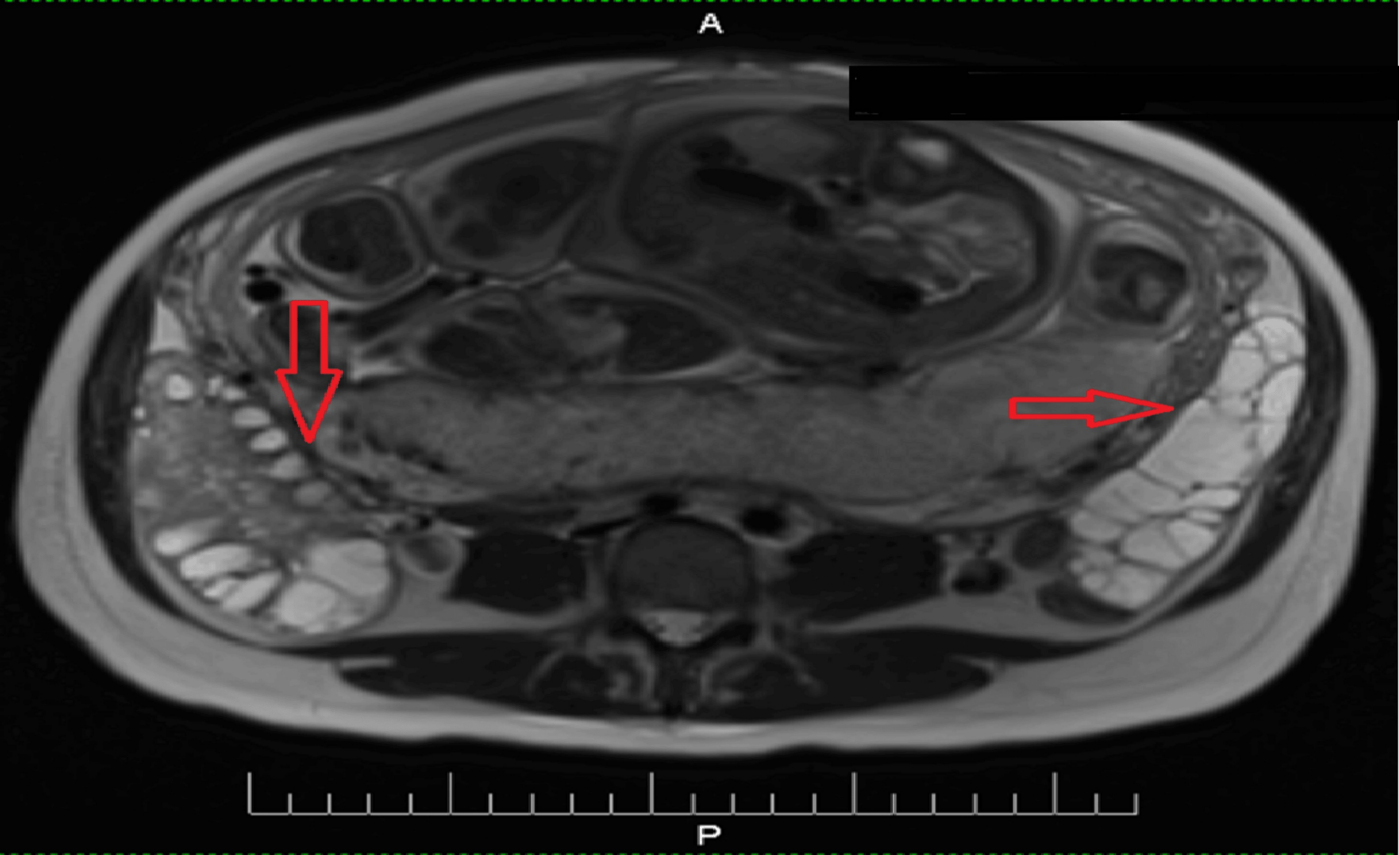
MRI image of enlarged ovaries A: anterior; P: posterior

An MRI was also performed. A single fetus was visualized in the cephalic position within the uterus. The placenta was attached to the posterior uterine wall. The urinary bladder was empty, and a catheter (drainage tube) was visible in the cavity. The right ovary measured 11.2 cm × 5.3 cm × 9.3 cm, with multiple cystic inclusions distributed peripherally, with a maximum diameter of 2.5 cm. A small amount of fluid was seen adjacent to the ovary. The left ovary consisted primarily of multiple cystic structures, with no visible solid tissue. The overall size (total size of cystic formations) was 11.4 cm × 4.1 cm × 9.1 cm. A small amount of fluid was also seen near the left ovary. No free air was seen in the abdomen or pelvis. The right ovarian vein was dilated, with a maximum diameter of 1.9 cm. No other significant findings were noted on MRI (Figures 2, 3).

No abnormalities were observed in the ovaries on ultrasound examination one month before this event. The patient was monitored in the emergency department. The general surgeon excluded appendicitis. The urologist decided to insert a urinary catheter, and the urine output was 500 mL; the severity of the pain decreased after the procedure. Since the laboratory findings and clinical presentation did not correspond to the diagnosis of ovarian torsion, the patient was admitted to the perinatology department for further observation.

Within a couple of hours, the patient began complaining of severe, constant abdominal pain. The patient was examined again. On abdominal palpation, the abdomen was soft, with tenderness and guarding in the ileocecal and hypogastric regions, separate from the uterine fundus. The uterus was enlarged, corresponding to approximately 36 weeks of gestation. The fundal height was 36 cm. The fetal lie was longitudinal, and the presentation was cephalic. No uterine tenderness or contractions were noted during the examination. The fetal heart rate was 145-150 bpm, and no uterine contractions were detected on CTG. No palpable masses were detected apart from the gravid uterus. Pasternatsky’s sign was negative bilaterally.

**Figure 4:**
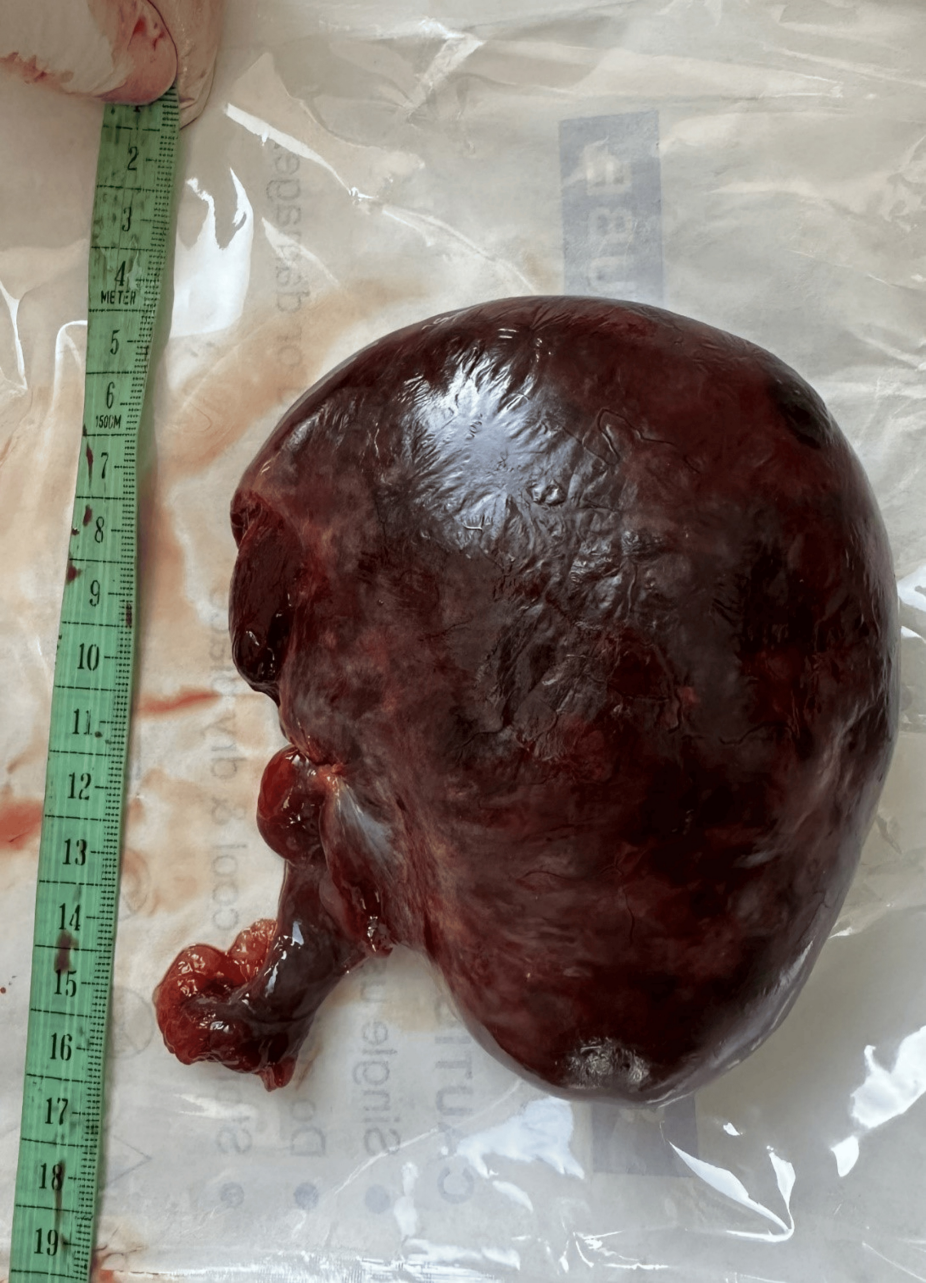
Right ovary after surgical removal

**Figure 5:**
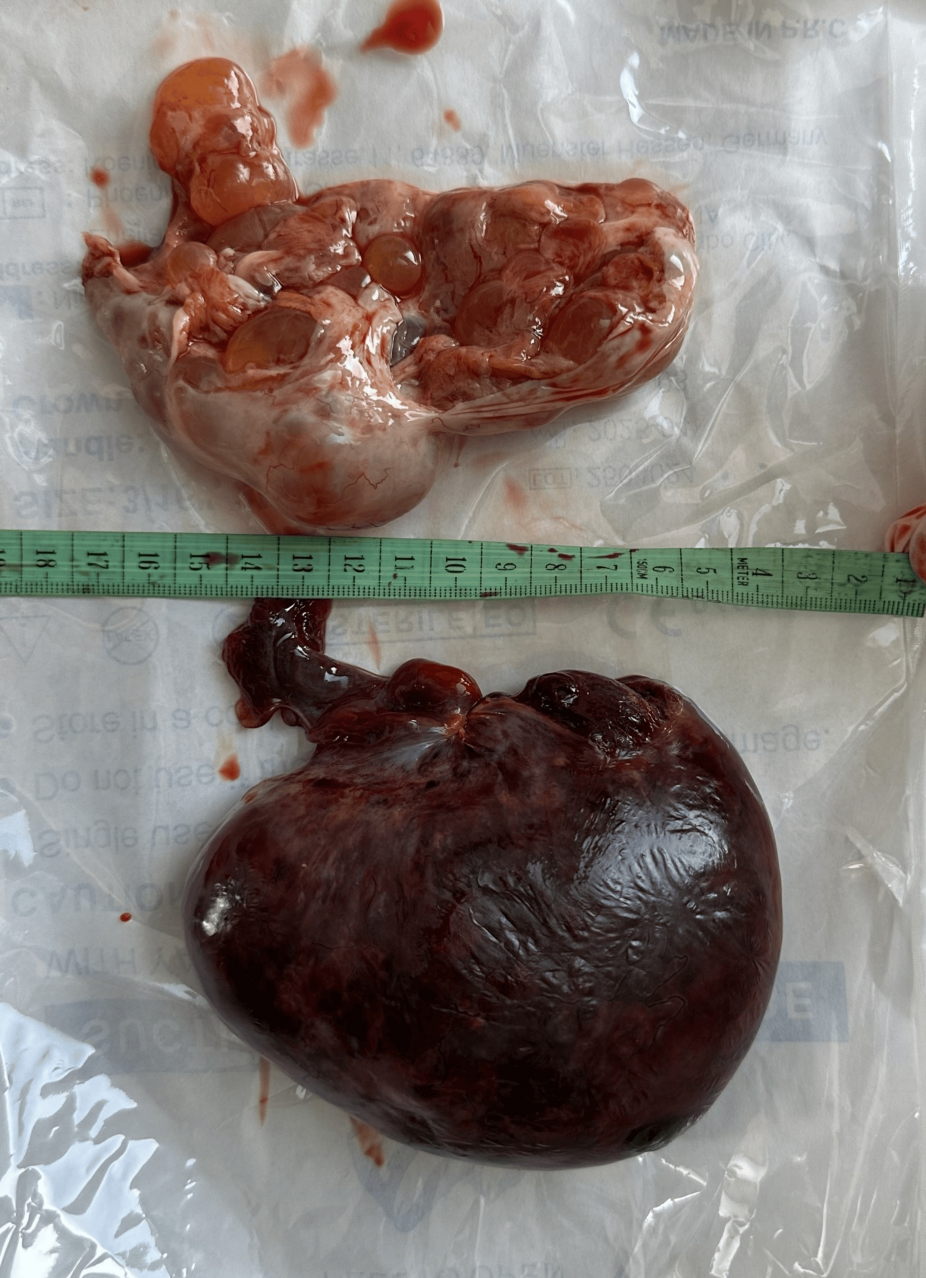
Left and right ovaries after surgical removal

She was diagnosed with an acute abdomen and ovarian torsion, and the decision was made to perform surgery: a C-section and a corresponding intervention on the ovary. The C-section was performed without complications. A live, preterm male fetus was delivered with a birth weight of 3250 g, a length of 49 cm, and an Apgar score of 8/8. After suturing the uterus, a revision of the abdomen was performed, which revealed bilaterally enlarged ovaries measuring approximately 12 cm in diameter. A bluish-black ovary was observed on the right side, which was completely twisted around its pedicle. The left ovary was almost entirely composed of cysts of various sizes, with only a small amount of normal ovarian tissue remaining. The decision was made to perform a right-sided adnexectomy and a partial ovarian resection on the left side. The surgery was completed without complications (Figures 4, 5).

**Figure 6:**
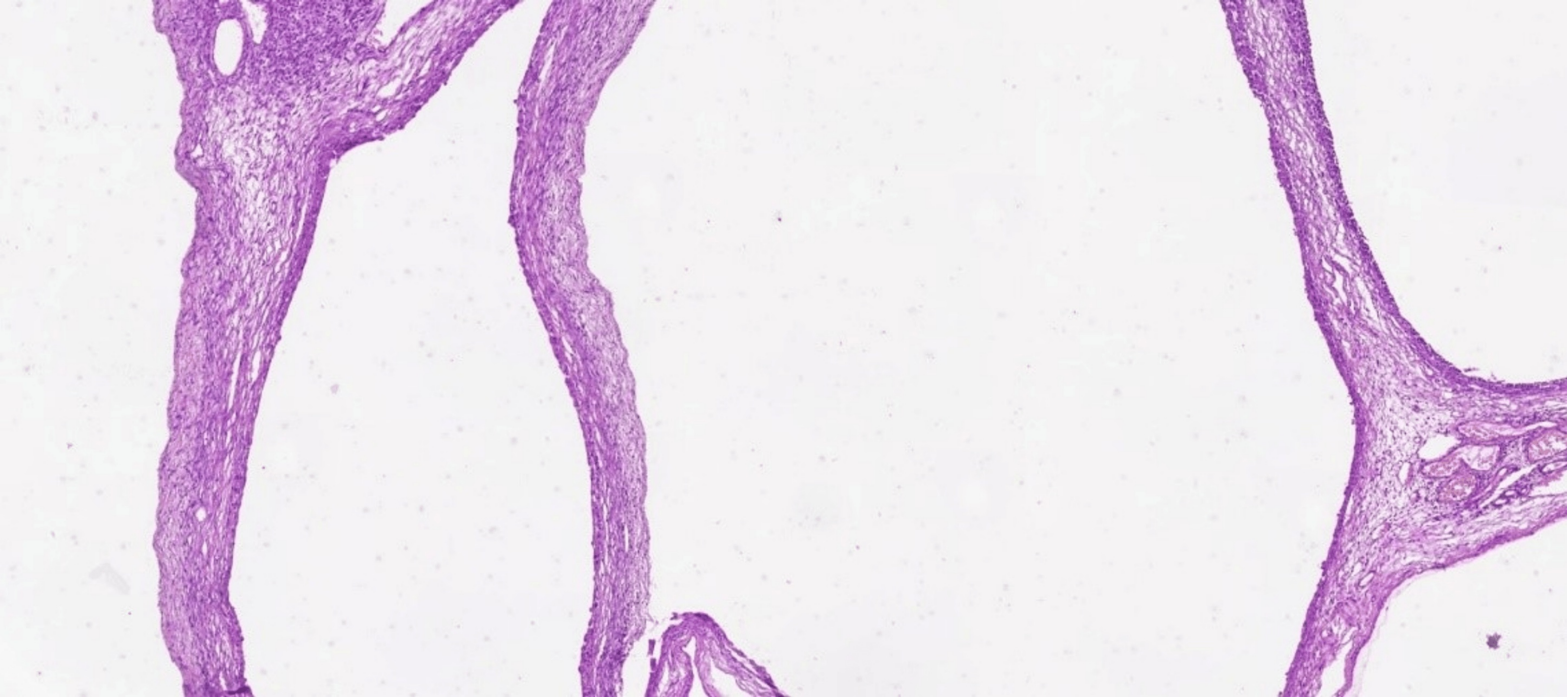
Histological image showing cyst walls of various sizes lined by fibrous tissue and theca cells Hyperemic blood vessels are visible within the wall (H&E stain, ×40). H&E: Hematoxylin and Eosin

**Figure 7:**
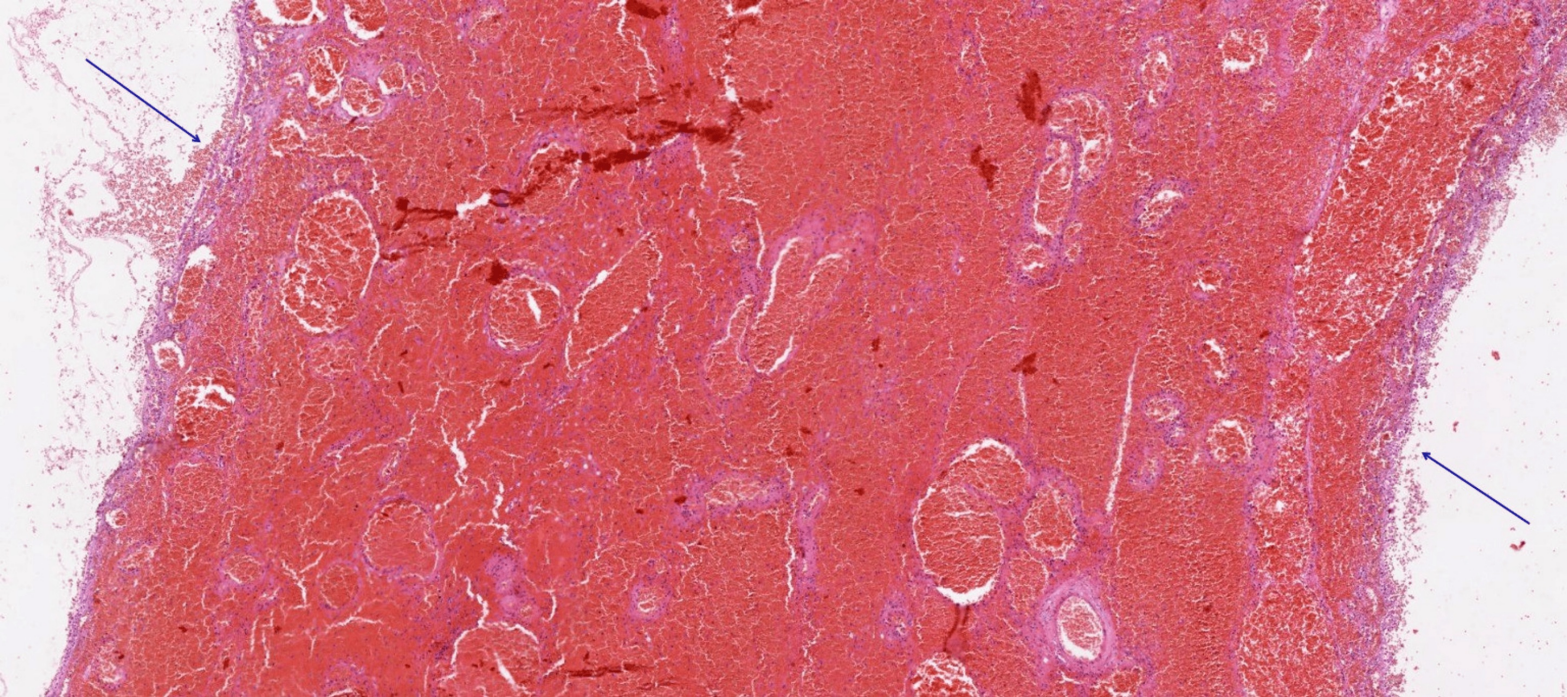
Histological image showing cysts lined by theca cells (arrows) Hemorrhage is visible within the cyst wall (H&E stain, ×40). H&E: Hematoxylin and Eosin

**Figure 8:**
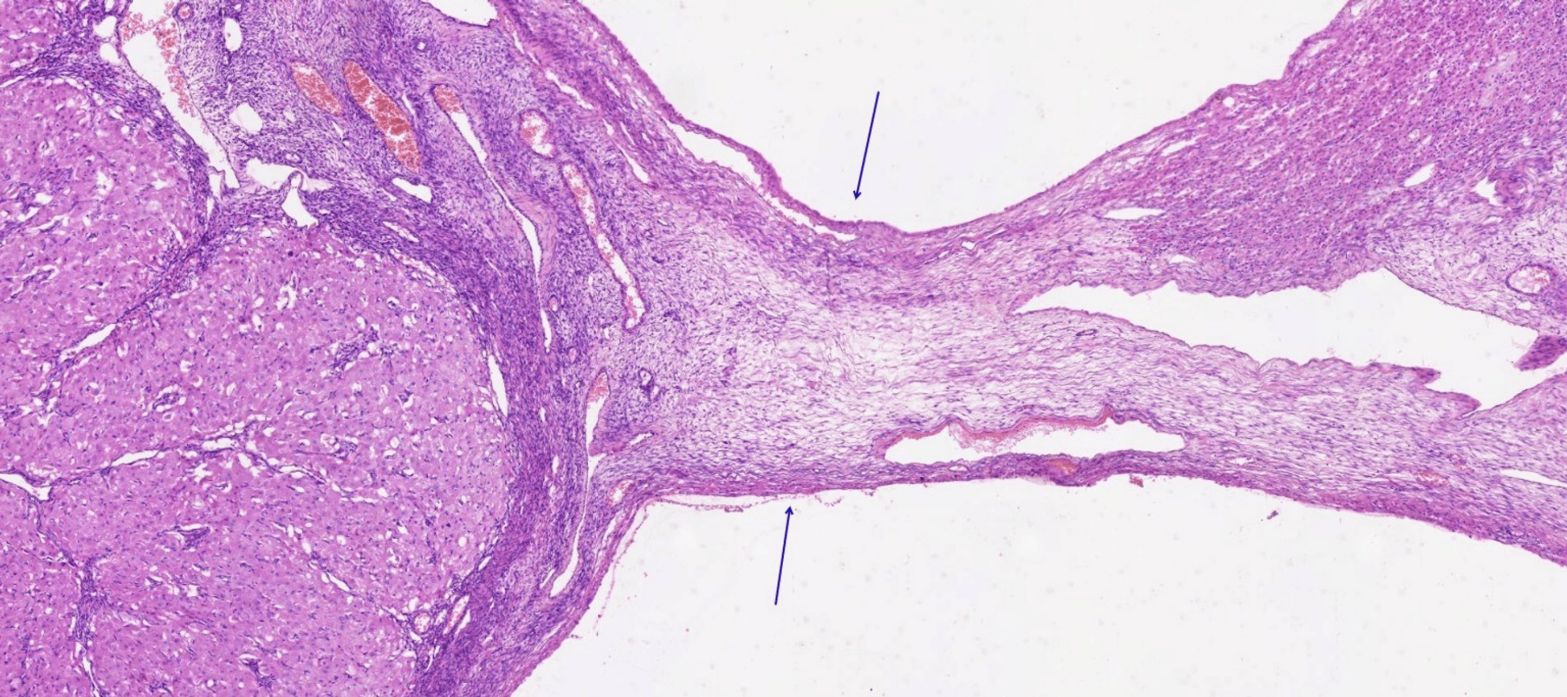
Fibrous cyst wall lined by theca cells (arrows) near a luteal cyst Near the luteal cyst (lower left corner), the image shows the fibrous cyst wall lined by theca cells (arrows). Hyperemic blood vessels are also visible (H&E stain, ×40). H&E: Hematoxylin and Eosin

The subsequent histomorphological evaluation revealed serous cysts with extensive hemorrhage in the right ovary. Serous cysts and a corpus luteum were identified in the left ovary (Figures 6, 7, 8).

## Discussion

Luteal cysts are functional ovarian cysts arising from the corpus luteum, which typically regress after 10-12 weeks of gestation. Luteal cysts occurring during advanced gestational ages are commonly associated with conditions such as gestational trophoblastic disease, multifetal gestation, and placentomegaly. The mechanism of luteal cyst development is related to human chorionic gonadotropin (hCG). hCG is a hormone composed of two subunits, α and β, joined together. The α subunit is common to other pituitary hormones (LH, FSH, and TSH), whereas the β subunit is unique to hCG and confers its specific biological activity. This activity primarily involves maintaining early pregnancy by stimulating the corpus luteum to produce progesterone. Sustained high levels of hCG, in forms such as the free β-subunit or the intact hormone, can cause enlargement of the corpus luteum, potentially leading to cyst formation.

The detection of luteal cysts in the third trimester is rare and suggests abnormal persistence or secondary enlargement of luteal tissue. As the cyst grows, the risk of ovarian or cyst torsion increases. These cysts are common in early pregnancy and usually resolve spontaneously by the second trimester [6].

Ovarian torsion is defined as the complete or partial rotation of the adnexa around its vascular axis or pedicle. Although the exact etiology remains unclear, predisposing factors include increased ovarian size, free mobility, and a long pedicle [2]. Emergency surgery during pregnancy for the management of a torsed or ruptured adnexal mass is uncommon (<5% of cases) and may lead to preterm delivery [7-10]. The risk of ovarian torsion increases approximately fivefold during pregnancy [11]. About 10%-25% of ovarian torsion cases occur in pregnant patients [12,13].

Torsion during the third trimester is rare, as the gravid uterus exerts a compressive effect that reduces the mobility of the ovarian pedicles [14,15]. The incidence of adnexal masses complicating pregnancy ranges from 0.05% to 2.4%, and approximately 1%-6% of these masses are malignant [7,16-18]. The majority of adnexal masses detected during pregnancy are benign simple cysts measuring less than 5 cm. Most represent functional ovarian cysts, such as follicular or corpus luteum cysts, that develop as part of normal ovarian activity. Nearly 70% of adnexal cystic masses identified during the first trimester resolve spontaneously by the beginning of the second trimester, which reflects the natural course of functional cysts [19].

Epithelial ovarian tumors account for about half of ovarian cancers diagnosed during pregnancy, whereas germ cell malignancies represent roughly one-third of cases. The remaining tumors consist of stromal tumors and other less common types [20].

According to the literature, luteal cysts are usually absent in the third trimester of pregnancy or, at least, do not increase in size. Therefore, luteal cyst torsion during the third trimester is rare. The presented case is noteworthy because it describes luteal cyst torsion in the third trimester of pregnancy. It should also be noted that the patient did not receive any pregnancy-supporting treatment.

## Conclusions

Ovarian torsion in the third trimester of pregnancy is a rare complication because of the decreased free space in the peritoneal cavity. Diagnosis is complicated due to the physiological changes observed during the third trimester of pregnancy. Physicians should always keep in mind that, in the absence of laboratory abnormalities and acute clinical signs of torsion, this complication can still occur. The low probability of this pathology does not exclude the possibility of ovarian torsion in the third trimester of pregnancy. Therefore, it should be considered by obstetricians in patients presenting with unexplained abdominal pain.
